# Lipid profiling of *C. elegans* strains administered pro-longevity drugs and drug combinations

**DOI:** 10.1038/sdata.2018.231

**Published:** 2018-10-23

**Authors:** Tesfahun Dessale Admasu, Krishna Chaithanya Batchu, Li Fang Ng, Amaury Cazenave-Gassiot, Markus R. Wenk, Jan Gruber

**Affiliations:** 1 Department of Biochemistry, Yong Loo Lin School of Medicine, National University of Singapore, Singapore, Singapore; 2 Department of Biochemistry, College of Medicine and Health Science, University of Gondar, Gondar, Ethiopia; 3 Science Division, Yale-NUS College, Singapore, Singapore; 4 Singapore Lipidomics Incubator, Life Sciences Institute, National University of Singapore, Singapore, Singapore

**Keywords:** Ageing, Lipidomics, Caenorhabditis elegans, Mass spectrometry

## Abstract

We report the effect of four lifespan modifying drugs and of synergistic combinations of these drugs on lipid profile in *Caenorhabditis elegans*. We employ ultra-high performance liquid chromatography-mass spectrometry (UHPLC-MS) to compare the abundance of lipid species in treated and control animals. Adult nematodes were treated with rapamycin, rifampicin, psora-4 and allantoin and combinations of these compounds and the resulting change in lipid profiles, specifically in those of triacylglycerol (TAG), phosphatidylcholine (PC) and phosphatidylethanolamine (PE) were determined. We quantified changes resulting from treatment with the drug combinations relative to untreated controls and relative to animals treated with each constituent single drugs. We further determined the dependence of changes in lipid profiles on genes known to affect lipid metabolism using strains carrying mutations in these pathways. In particular, we determined lipid profiles in a genetic model of caloric restriction (*eat-2*), a strain lacking homolog of TGFβ (*daf-7*) and in a strain lacking the SREBP*/sbp-1* transcription factor.

## Background & Summary

Ageing is a complex biological phenomenon and a major risk factor for many chronic diseases^1–4^. Although there are many genes and pathways known to modulate ageing and longevity, the ultimate mechanism by which these pathways affect organismal longevity is not fully understood. Insulin-like growth factor (IGF), mechanistic target of rapamycin (mTOR), AMP-activated protein kinase (AMPK) and sirtuins are among the best-characterized pathways known to regulate longevity and affect ageing trajectories in model organisms^5,6^. An increasing number of drugs and drug-like molecules, including human drugs such as metformin and rapamycin, have been identified to target pathways involved in ageing and longevity and these compounds are known to extend lifespan and health span in model organisms^7–10^. The observation that such benefits are conserved over large evolutionary distances suggests that similar pro-longevity and health span effect may exist in humans^8^. Indeed, there is some evidence that conserved longevity and ageing pathways modulate age-dependent decline and disease in humans and that drugs known to target these pathways may have benefit in ageing humans^11^.

However, compared to genetic mutations targeting known ageing pathways, the typical effect sizes of adult-onset drug interventions are relatively small, even in short-lived model organisms^12^. Interestingly, it has been shown that double and triple genetic mutations can lead to synergistic lifespan extension benefits by leveraging beneficial interactions between several ageing pathways^13,14^. There is a growing interest in translating such beneficial pathway synergies into combinatorial drug treatments^15,16^. Such combinatorial interventions have the potential to achieve increased efficacy at a lower dose of the individual compounds, thereby potentially reducing side effects and increasing safety margins. However, a major challenge in identifying beneficial drug interactions in the context of ageing is that a large number of possible combinations to be screened and the fact that lifespan studies, even in model organisms, are time consuming and expensive. Without carrying out combinatorial lifespan studies, however, there is currently no *a priori* method to determine whether a given drug combination interacts and whether any such interactions will be toxic, beneficial or synergistic during ageing. While computational approaches aimed at predicting such interactions exist, at least in the case of lifespan modulation they remain to be experimentally validated^13^. There is, therefore, interest in developing and leveraging “-omics” technologies (lipidomics, proteomics, transcriptomics) to facilitate identification of potentially beneficial drug synergies or of detrimental drug interaction based on cheaper short-term biomarker studies. In our study^17^, we, therefore, determined transcriptomics and lipidomics profiles of drugs and drug combinations with the aim of investigating features and signatures associated with beneficial drug synergies.

As part of this study, we carried out extensive lipid profiling in wild-type (WT) and mutant nematodes treated with drugs and drug combinations. Here we report complete lipid profiles of wild-type N2, *eat-2, sbp-1* and *daf-7* mutant nematodes treated with lifespan extending drug combinations.

## Methods

These methods are expanded versions of descriptions in our related work^17^ supplemented with details where necessary. Additional figures, MS scans, can be found at MetaboLights (Data Citation 1) and Figshare (Data Citations 2, 3, 4, 5).

**Figure 1:**
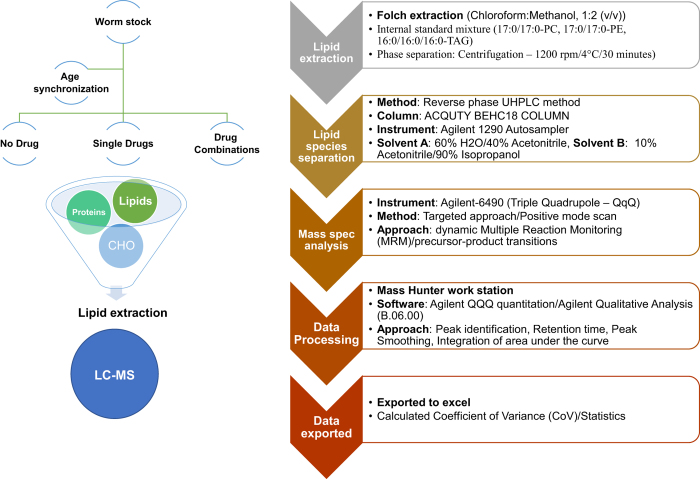
Overview flowchart of sample preparation, extraction, data generation and analysis. Age synchronized young adult nematodes were treated with individual drugs, drug combinations or vehicle for two days, starting at day four of age, followed by harvesting and lipid extraction at day six. Lipid species were separated by reverse phase UPLC and subjected to MS analysis. Data were processed using the Mass Hunter Workstation software package (Agilent, version B.06.00) and exported to excel for statistical analysis.

We treated age synchronized young adult wild-type N2 nematodes with rapamycin, rifampicin, allantoin, psora-4 or their synergistic double or triple combinations; rapamycin+rifampicin, rifampicin+posra-4, rapamycin+rifampicin+allantoin or rifampicin+psora-4+allantoin (Fig. 1). We then determine changes in lipid profiles in treated wild-type animals as well as *eat-2, daf-7* and *sbp-1* mutants exposed to these synergistic combination treatments. We determined changes in lipid species of Triacylglycerol (TAGs), phospholipids (Phosphatidylcholine (PCs) and Phosphatidylethanolamine (PEs)) using LC-MS (Fig. 1). Here we present the detailed protocol used for this lipid profiling in *Caenorhabditis elegans* (*C. elegans)* and the associated data description.

### Nematode growth medium (NGM) agar plate preparation

**Step 1:** 1.5 g NaCl, 8.5 g bacto agar, and 1.25 g peptone were dissolved in 487.5 ml H_2_O for a final volume of 500 ml.

**Step 2:** The NGM agar solution was then autoclaved and kept in a water bath until the temperature had dropped to 55 °C.

**Step 3:** 12.5 ml of potassium phosphate buffer (132 ml K_2_HPO_4_ (1 M) with 868 ml KH_2_PO_4_ (1 M), and 0.5 ml of 1 M MgSO_4_, 1 M CaCl_2_, cholesterol (5 mg/ml in ethanol), streptomycin (200 mg/ml) and 250 μM of 5'-fluorodeoxyuridine (FUdR) were added to the NGM agar solution.

**Step 4:** The NGM agar was then aliquoted into smaller volumes depending on the experimental conditions and drugs or vehicle were added to the final concentration of each drug.

**Step 5:** 40 ml of the NGM agar solution with or without drugs was then poured each into 145 mm Petri dishes and allowed to solidify.

**Step 6:** Frozen bacterial stocks, pre-concentrated to 10^10^ colony forming unit per ml (CFU/ml) were thawed and drug or vehicle was added to these bacterial stocks to the final concentration.

**Step 7:** 3 ml of bacteria were seeded onto each NGM agar plate in droplets and allowed to dry. Plates were used directly when the nematodes are ready otherwise stored at 4 ^o^C for a maximum of two weeks before use.

### Bacterial Food preparation

**Step 1:** 25 g of Luria broth (LB) was dissolved in 1 L water, and autoclaved. LB was cooled and used directly for the next step or stored at 4 ^o^C for a maximum of one month.

**Step 2:** 1 ml of 200 mg/ml streptomycin was added per 1 L of LB and 5 ml of LB with streptomycin was aliquoted into five 50 ml tubes.

**Step 3:** A single colony of bacteria was taken from a stock bacterial plate and used to inoculate a starter culture containing 5 ml of LB in a 50 ml falcon tube. One tube was handled the same but not inoculated and used as negative control.

**Step 4:** Bacterial starter cultures were then grown at 37 ^o^C with shaking (180 rpm) for 16 h.

**Step 5:** 5ml of starter culture was used to inoculate 1 L of LB broth in the 2 L flask.

**Step 6:** The bacterial cultures were incubated at 37 ^o^C with shaking (180 rpm) for 18–20 h.

**Step 7:** Bacterial culture was centrifuged at 5000 g for 10 min to pellet OP50-1. The supernatant was removed and discarded.

**Step 8:** Bacterial pellets were collected and re-suspended and diluted to 10x, 100x, and 1000x in M9 buffer (3 g KH_2_PO_4_, 6 g Na_2_HPO_4_, 5 g NaCl, 1 ml 1 M MgSO_4_, H_2_O to 1 litre. Sterilize by autoclaving). The concentration was determined using a UV-Visible spectrophotometer. The bacteria were diluted to final concentration of 10^10^ CFU/ml.

**Step 9:** Bacterial food stock was aliquoted into small volumes of 50 ml falcon tube and stored at −80 ^o^C.

### Worms age synchronization

**Step 1**: Worms were chunked onto a pre-seeded 10 cm NGM plate and allowed to grow up to 2–3 days until large numbers of eggs and gravid adults were apparent on the plate.

**Step 2**: 7 mL of M9 was poured onto the plate and gently swirl it to dislodge the worms and the worms were transferred to 15 mL tube.

**Step 3**: 2 ml of bleach (10% alkaline hypochlorite) and 1 ml of 5 M NaOH was added. The tube was mixed gently by inverting for 7–8 min.

**Step 4**: Synchronized eggs were centrifuged for 1 minute at low g (2.5 g) to pellet them.

**Step 5**: The M9 was aspirated without disturbing the egg pellet.

**Step 6**: About 10 mL of M9 was added to the tube and mixed well.

**Step 7**: Tube was centrifuged again at 2.5 g for one minute.

**Step 8**: Most of the M9 was aspirated without disturbing the worm pellet.

**Step 9**: Steps 6–8 were repeated at least two times.

**Step 10**: Pellet was suspended by 0.5 ml M9 and 10 μl of the pellet was transferred to a microscope slide for inspection. Number of eggs in several drops was counted and a total number of eggs was estimated from these counts.

**Step 11**: Eggs were seeded to fresh NGM plate.

**Step 12**: After three days, adult nematodes are ready for drug treatment.

### Culturing of *C. elegans*

**Step 1:** Bristol wild-type N2, the genetic caloric restriction *eat-2* mutant DA1116, TGFβ/ *daf-7* mutant CB1372 and SREBP/ *sbp-1* mutant CE541 were obtained from the *C. elegans* genetics Centre (CGC) and used for this study.

**Step 2:** Wild-type N2, DA1116 and CE541 were cultured at 20 ^o^C for all experiments. Temperature sensitive strain CB1372 was cultured at 15 ^o^C until adulthood and then transferred to 20 ^o^C at the age of day 4.

**Step 3:** For all experiments, nematodes were grown and maintained on solid NGM agar plates using *E. coli* OP50-1 bacteria as a food source.

**Step 4:** NGM agar plates were prepared as mentioned earlier; 145 mm Petri dishes were used. To each 145 mm petri dish, 45 ml of NGM agar with or without drug was added onto each plate.

**Step 5:** For drug treatment, all NGM agar plates were prepared from the same batch of NGM agar. Treatment plates and bacterial food were supplemented with the respective drugs or vehicle (controls).

**Step 6:** 250 μM of FUdR was added to the NGM medium to prevent the synchronized population of *C. elegans* from reproducing.

**Step 7:** About 1000 day 4 age-synchronized young adult nematodes were transferred to each plate. The nematodes were cultured at 20 ^o^C.

**Step 8:** Nematodes were collected by washing plates with M9 buffer after 48 h of drug treatment. The nematodes were washed several times to remove the bacteria, eggs, and larvae.

**Step 9:** 5% glycerol was added to each nematodes pellet and keep at −80 ^o^C until they are ready for lipid extraction.

### Drug treatment overview

**Step 1:** Rapamycin, Rifampicin, Allantoin, Psora-4 and their synergistic double and triple combinations were used in this study.

**Step 2:** Stock solutions of each drug were prepared by dissolving solid drug stock in Dimethyl Sulfoxide (DMSO).

**Step 3:** Since Psosra-4 is light sensitive, we keep Psora-4 solution in dark or covered with aluminum foil.

**Step 4:** Appropriate volume of stock solutions of each drug were added to both the NGM medium and bacterial food to get the final concentration of each drug.

**Step 5:** An equal amount of DMSO was added to the control plates.

### Reagents

For 100mL NGM:

1. 100 μL 100% Cholesterol
2. 100 μL 1 M CaCl_2_
3. 100 μL 1 M MgSO_4_
4. 2.5 mL Phosphate Buffer (pH=6.0)
5. Stock solution of drugs
6. 100 μL 100 mg/mL FUDR (if required)
7. 100 μL streptomycin

### Drug Treatment Protocol

**Step 1:** Autoclave NGM (100 mL).

**Step 2:** Heat water bath to 55 ºC.

**Step3:** Once NGM has cooled to 55 ºC, put NGM into a water bath for 15 min and add the above reagents.

**Step 4:** Add the drug or drugs and mix the NGM by swirling the bottle.

**Step 5:** Make DMSO control plates in parallel. Use the above protocol but instead of drug add an equal amount of DMSO (final concentration should be less than 0.2%).

**Step 6:** For Lipidomics assay: Transfer young adult nematodes, 60 h post-bleaching, on drug and control plates and continue treatment for 48 h.

**Step 7:** Collect nematodes by washing the plates with M9 buffer.

**Step 8:** Store the sample at −80 ^o^C overnight for a maximum of 24 h. Lipid was extracted after 24 h of molting.

### Reagents and instruments for lipid extraction

1. LC-MS Grade Organic Solvents: Chlorofrom (CHCl_3_), Methanol (MeOH), Isopropanol (IPA), Acetonitrile (ACN) (Fisher Chemical, Belgium)
2. Ammonium formate (10 mM final concentration)
3. Butylated hydroxytoluene (BHT)
4. Reverse phase ZORBAX Eclipse Plus C18 column (2.1×50 mm, 1.8-Micron)
5. Instrument: Agilent 1290 Infinity LC system -6490 Triple Quadrupole
6. Lipid standards (1,2-diheptadecanoyl-sn-glycero-3-phosphocholine (17:0/17:0-PC), 1,2-diheptadecanoyl-sn-glycero-3-phosphoethanolamine (17:0/17:0-PE) and [1,1,2,3,3-pentadeuterio-2,3-di(hexadecanoyloxy)propyl] hexadecanoate (d5-16:0/16:0/16:0-TG) – Purchased from Avanti polar lipids
7. Bead beater (Precellys 24 tissue homogenizer, Bertin instruments, France)

### Lipid extraction for LC-MS analysis

**Step 1:** For each condition, a pellet containing 2000–3000 nematodes was collected, washed with M9 buffer and transferred to 2 mL polypropylene tubes containing 250 μl lysis buffer (20 mM Tris-HCl pH 7.4, 100 mM NaCl, 0.5 mM EDTA, 5% glycerol) and incubated on ice for 15 min followed by homogenization using a bead beater (Precellys, France) maintained at 4 °C.

**Step 2:** Lipids extraction from the lysed samples was carried out by Folch’s extraction^18^. Briefly, 800 μl of ice-cold chloroform-methanol (1:2; volume/volume (v/v)) was added to the homogenates and vigorously vortexed for 1 minute followed by shaking at 4 °C at 1200 RPM for 30 minutes using a thermomixer (Eppendorf, Hamburg, Germany). In order to minimize oxidation during extraction, 0.5% butylated hydroxytoluene (BHT) was added to the organic solvents.

**Step3:** For method validation and to control lipid-class dependent differences in extraction and ionization, at the time of extraction samples were spiked with known amounts of internal standards (1,2-diheptadecanoyl-sn-glycero-3-phosphocholine (17:0/17:0-PC), 1,2-diheptadecanoyl-sn-glycero-3-phosphoethanolamine (17:0/17:0-PE) and [1,1,2,3,3-pentadeuterio-2,3-di(hexadecanoyloxy)propyl] hexadecanoate (d5-16:0/16:0/16:0-TAG) (purchased from Avanti polar lipids, Alabaster, AL, USA) corresponding to each lipid class.

**Step 4:** Upon centrifugation, the organic phase was transferred to a fresh centrifuge tube and dried under vacuum using a vacuum concentrator (SpeedVac, Thermo Savant, Milford, USA).

**Step5:** The dried lipid extract was reconstituted in 100 μl methanol and kept at −80 °C until the MS analysis was performed.

### Lipid analysis by Mass spectrometry

**Step 1:** Sample (5 μl) was injected using an Agilent 1290 Infinity LC system (Agilent Technologies, Santa Clara, CA) coupled to a triple quadrupole (QqQ) instrument (Agilent 6490) for MS analysis.

**Step 3:** Chromatographic separation was achieved on the LC system equipped with a ZORBAX Eclipse Plus C18 column (2.1×50 mm, 1.8-Micron). Separation of the molecular species was done using a gradient elution. Solvent A was acetonitrile/H_2_O (60:40 v:v) with 10 mM ammonium formate, while solvent B was isopropanol/acetonitrile (90:10) containing 10 mM ammonium formate.

**Step 4:** The flow rate was set to 0.4 mL/min and the column temperature was 45 °C. Solvent B was set at 40% at injection and increased linearly to 100% in 7 min, retained at this value for 2 min, decreased back to 40% in one minute and then retained there until the end of the gradient by 14 min. The eluent was directed to the ESI source of the mass spectrometer operated in the positive ion mode.

**Figure 2:**
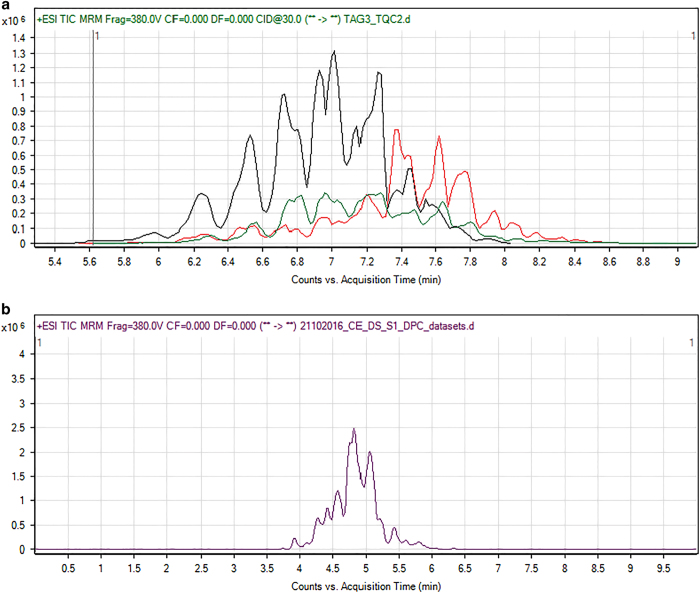
Representative chromatogram of TAG and PC lipid species with retention time. (**a**) Total Ion Chromatograms (TIC) of triacylglycerols detected by three different dynamic multiple reaction monitoring (dMRM) methods. Each method monitored a specific set of TAG molecular species, hence the variations in retention times between the three TICS (red, black and green). (**b**) A representative chromatogram showing LC separation of phosphatidylcholines from *C. elegans* lipid extracts. Here the x-axis is the total acquisition time (0 min to 11 min) that shows the retention time of molecular species monitored for while the y-axis represents the total ion count.

**Step5:** Lipids were ionized by ESI and each individual lipid molecular species was analysed using a targeted dynamic multiple reaction monitoring (dMRM) approach measuring over several unique transitions with known precursor/product mass-to-charge ratio (m1/m3) and retention times (Fig. 2a,b).

**Step6:** The MS conditions were as follows. For ESI: gas temperature, 300 °C; gas flow, 10 l/minutes; sheath gas temperature, 350 °C; sheath gas flow, 8 l/minutes; and capillary voltage, 3500 V.

**Step7:** The resolution of the instrument is of about 1,000 at *m/z* 760 and a mass accuracy of >200 ppm. The minimum dwell time used was 19 ms while the run time was 14 min. MRM method was used where Q1 and Q3 were set to pass molecularly distinctive precursor and productions, whereby collisions were induced in Q2 using N_2_. A complete list of the MRMs measured in this study and raw data are available as tables (Tables 1, 2 and TAGs lipid species (Data Citation 2)) and MS scans in *MetaboLights* (Data Citation 1).

**Figure 3:**
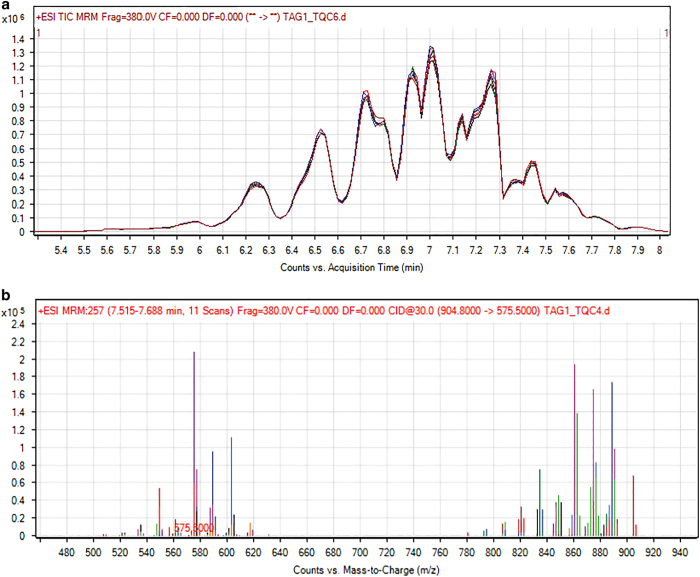
Representative technical quality control and MS spectrum of TAG. (**a**) Assessing data quality by overlaying six TICs of the technical quality control sample analyzed at regular intervals between the experimental samples. (**b**) A representative MS spectrum extracted from one of the technical quality control sample showing TAG species counts between 450 m/z and 950 m/z.

**Step 8:** Each sequence run was set up with three Blank extraction runs at the beginning followed by three Quality control (QC) runs. Next, the experimental samples were measured with a QC measurement at regular intervals (every 10 experimental samples) (Fig. 3a,b).

### Code availability

No custom made codes have been used in this study.

## Data records

LC-MS raw datasets

Uploaded to *MetaboLights* (Data Citation 1).

**Mutants_PC datasets**=All PC MS scans pertaining to mutant strains (*eat-2, daf-7,* and *sbp-1*) with and without drug treatments.

**Mutants_PE datasets**=All PE MS scans pertaining to mutant strains (*eat-2, daf-7,* and *sbp-1*) with and without drug treatments.

**Mutants_TAGs datasets**=All TAGs MS scans pertaining to *eat-2* with and without drug treatments.

**N2_PC datasets**=All PC MS scans pertaining to wild-type N2 nematodes with and without drugs and drug combinations treatments.

**N2_PE datasets**=All PE MS scans pertaining to wild-type N2 nematodes with and without drugs and drug combinations treatments.

**N2_TAGs datasets**=All TAGs MS scans pertaining to wild-type N2 nematodes with and without drugs and drug combinations treatments.

## Technical Validation

Technical validation of the LC-MS method was achieved by employing non-endogenous or labeled lipid standards for each class. These were spiked at known concentrations (2 nmol/μl) into samples. Spiking was done during the two-phase liquid-liquid extraction. The reference standards that were used in this study were 1,2-diheptadecanoyl-sn-glycero-3-phosphocholine (17:0/17:0-PC), 1,2-diheptadecanoyl-sn-glycero-3-phosphoethanolamine (17:0/17:0-PE) and [1,1,2,3,3-pentadeuterio-2,3-di(hexadecanoyloxy)propyl] hexadecanoate (d5-16:0/16:0/16:0-TAG).

To ensure the accuracy and reproducibility of MS generation and to facilitate interpretation of differences in lipid species, technical coefficients of variance (CoV) were calculated for all the MRM transitions measured in the pooled quality control (QC) sample run at regular intervals (once in between every 10 experimental samples) (Fig. 3a,b). The technical CoV is defined as the variation due to the combined pre-processing of samples (process CoV) and analytical variation (instrument CoV). These values were calculated from normalized data (to spiked internal standard) for the pooled QC samples (n=6) as the ratio of standard deviation (S.D) to average intensity, multiplied by 100. Only those MRM transitions that passed the QC test (CoV&lt;25%) were included in the later analysis of actual samples. In total, 361 MRMs of the 434 TAG species measured, 35 of the 39 MRMs pertaining to different PC molecular species and 26 of the 31 PE MRMs passed the QC and were included in the analysis (see Data Citations 3, 4, 5).

### Data processing

**Step 1:** High-resolution LC-MS data obtained on the 6490 QqQ Mass spectrometer were subjected to data processing on a Mass Hunter software (Agilent) workstation (version B.06.00) (Refer to MS data in *MetaboLights* Data Citation 1).

**Step 2:** The identification of a particular species was based on accurate mass and/or retention times (RT). Signal intensities of the targeted species were compared with the intensities from the spiked internal standards and the retention times for the various classes were matched (Tables 1, 2), (Fig. 2a,b).

**Figure 4:**
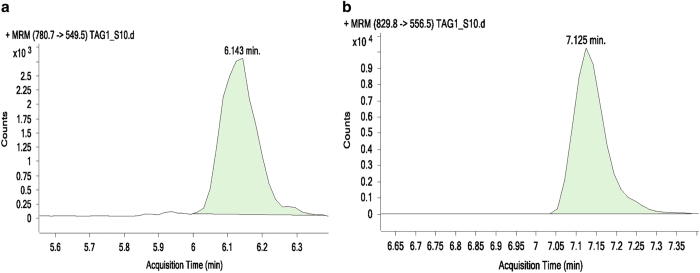
Extracted ion chromatograms (EIC) for individual TAG MRM transitions, displayed in the Agilent QQQ quantitative analysis software. (**a**) EIC for 45:1–13:0-TAG and (**b**) EIC for 48:0-16:0-TAG. Varying retention times can be observed with 45:1–13:0-TAG elution starting at 6:00 min while that of 48:0-16:0-TAG at 7:00 min due to the difference in hydrophobicity.

**Step 3:** Data processing, included peak smoothing and integration of areas under the curves for each ion measured. The processed data were exported to excel and normalized to the internal standard (Fig. 4a,b) (Data Citations 3, 4, 5).

**Step 4:** Fold changes were measured by comparison of the different conditions to the control sample and finally the Student’s t-test was performed to determine whether differences between the samples were statistically significant. (p < 0.05 was considered statistically significant).

### Summary of values compiled

**Analysis by averaging number of carbons and double bonds of phospholipid species** - The effect of the single drugs and the synergistic drug combinations on the degree of unsaturation in glycerophospholipids, mainly PC and PE, was assessed by pooling all those molecular species with an equal degree of unsaturation independent of chain length (at the *sn-1* and *sn-2* positions) (Phosphatidylethanolamine (PE) raw data and Phosphatidylcholine (PC) raw data) (Data Citations 3, 4, 5).

**Analysis of TAGs by combining acyl chain lengths** - We specifically analyzed the distribution of the combined chain length of the TAG molecular species by measuring their normalized abundances. The number of carbons in each of the three different fatty acyls (independent of the degree of unsaturation) were first summed up to get the total chain length (carbon sum composition). Next, abundances of TAG chain lengths containing between 51 and 56 carbons were pooled together while those that contained more than 56 carbons pooled separately (Triacylglycerides (TAGs) raw data) (Data Citation 5).

### Drug and Drug combination abbreviations

Rap=Rapamycin

Rif=Rifampicin

Pso=Psora-4

RifPso=Rifampicin + Psora-4

RapRif=Rapamycin + Rifampicin

RRA=Rapamycin + Rifampicin + Allantoin

RPA=Rifampicin + Psora-4 + Allantoin

## Usage Notes

These lipidomics profiles will be useful at least three different types of analyses. First, we provide reference lipid profiles for N2 wild-type, *eat-2, daf-7* and *sbp-1* mutant strains. N2 is the standard reference strain while *eat-2* is a long-lived strain thought to model calorie restriction. The *daf-7* strain lacks a *C. elegans* homolog of TGFβ and is long-lived. Finally, *sbp-1* (SREBP-1 in humans) is a major transcription factor upstream of several classes of enzymes involved in lipid metabolism. These data will, therefore, be of interest to researchers interested in the effects of CR, TGFβ or genes downstream of SREBP-1 on lipid metabolism. In addition, we provide lipid profiles for these mutant strains and wild-type controls following treatment with several lifespan-extending drugs and drug combinations. There is growing interest in drug synergies/interactions as a way to design efficacious interventions in the context of ageing. Our best synergistic drug combinations result in lifespan extension effects and health span benefits that are larger than those of any previously reported in the literature. The data characterizing the effects of these drugs and drug combinations on the lipid profiles should, therefore, be of interested to researchers exploring novel lifespan interventions based on drug synergies. The lipid profiles for single drugs, synergistic and non-synergistic combinations will help further analysis and understanding of features of drug synergy. Finally, combining this lipid profile with the transcriptional analysis that we have run in parallel^17^ should aid the development of strategies to identify drug interactions and synergy based on the multi-omics (transcriptomics and lipidomics in this context) profiles–without the need of time-consuming combinatorial lifespan studies.

## Additional information

**How to cite this article**: Admasu, T.D. *et al*. Lipid profiling of *C. elegans* strains administered pro-longevity drugs and drug combinations. *Sci. Data*. 5:180231 doi: 10.1038/sdata.2018.231 (2018).

**Publisher’s note**: Springer Nature remains neutral with regard to jurisdictional claims in published maps and institutional affiliations.

## Supplementary Material



## Figures and Tables

**Table 1:** PC lipid species depicts all the lipid species that were monitored for.

PC mol.species	Transition (m1/m3)	Retention time
PC 28:1	676.5–>184.1	1.4
PC 28:2	674.5–>184.1	4.6
PC 29:0	692.5–>184.1	3.1
PC 29:1	690.5–>184.1	3.9
PC 30:1	704.5–>184.1	4.2
PC 31:1	718.5–>184.1	4.0
PC 32:0	734.6–>184.1	4.4
PC 32:1	732.6–>184.1	4.1
PC 32:2	730.5–>184.1	3.8
PC 32:3	728.5–>184.1	3.6
PC 33:1	746.5–>184.1	4.3
PC 34:1	760.6–>184.1	4.4
PC 34:2	758.6–>184.1	4.2
PC 34:3	756.6–>184.1	3.9
PC 34:4	754.5–>184.1	3.7
PC 34:5	752.5–>184.1	3.6
PC 35:1	774.5–>184.1	4.6
PC 36:0	790.5–>184.1	4.7
PC 36:1	788.6–>184.1	4.8
PC 36:2	786.6–>184.1	4.6
PC 36:3	784.6–>184.1	4.3
PC 36:4	782.6–>184.1	4.0
PC 36:5	780.6–>184.1	3.9
PC 36:6	778.5–>184.1	3.6
PC 38:0	818.5–>184.1	3.9
PC 38:1	816.5–>184.1	3.7
PC 38:2	814.6–>184.1	4.9
PC 38:3	812.6–>184.1	4.6
PC 38:4	810.5–>184.1	4.3
PC 38:5	808.6–>184.1	4.1
PC 38:6	806.6–>184.1	4.0
PC 38:7	804.6–>184.1	3.7
PC 40:0	846.5–>184.1	5.4
PC 40:1	844.5–>184.1	3.6
PC 40:4	838.6–>184.1	4.7
PC 40:5	836.6–>184.1	3.9
PC 40:6	834.6–>184.1	4.3
PC 40:8	830.6–>184.1	3.8
PC 42:0	874.5–>184.1	3.2
PC 42:1	872.5–>184.1	4.1
PC 44:1	900.5–>184.1	4.7
PC 44:2	878.5–>184.1	4.7
PC 28:0	678.5–>184.1	2.8
MRM transitions and retentions time for the corresponding individual lipid species are also listed out.		

**Table 2:** PE lipid species depicts all the lipid species that were monitored for.

PE mol.species	Transition (m1/m3)	Retention time
PE 28:1	634.5–>493.5	1.9
PE 30:1	662.5–>521.5	3.8
PE 30:2	660.5–>519.5	3.6
PE 32:0	692.5–>551.5	4.4
PE 32:1	690.5–>549.5	4.2
PE 34:0	720.6–>579.5	4.9
PE 34:1	718.5–>577.5	4.6
PE 34:2	716.5–>575.5	4.3
PE 34:3	714.5–>573.5	4.1
PE 34:4	712.5–>712.5	4.1
PE 34:5	710.5–>710.5	1.1
PE 35:1	732.6–>591.5	5.1
PE 35:2	730.5–>589.5	4.5
PE 36:0	748.6–>607.6	4.9
PE 36:1	746.6–>605.6	4.9
PE 36:2	744.6–>603.5	4.7
PE 36:3	742.5–>601.5	4.4
PE 36:4	740.5–>599.5	4.2
PE 36:5	738.5–>597.5	4.1
PE 38:0	776.5–>635.5	4.0
PE 38:3	770.6–>629.6	4.8
PE 38:4	768.6–>627.5	4.4
PE 38:5	766.5–>625.5	4.4
PE 38:6	764.5–>623.5	4.1
PE 40:0	804.5–>663.5	5.5
PE 40:2	800.5–>659.5	5.3
PE 40:4	796.6–>655.6	4.8
PE 40:5	794.6–>653.6	4.8
PE 40:6	792.6–>651.5	4.4
PE 40:7	790.5–>649.5	4.2
PE 44:2	856.5–>715.5	5.8
PE 28:0	636.5–>495.5	3.8
MRM transitions and retentions time for the corresponding individual lipid species are also listed out.		
